# Association of estimated glucose disposal rate with all-cause and breast cancer-specific mortality in US breast cancer survivors: a population-based study

**DOI:** 10.1186/s12885-025-15340-0

**Published:** 2025-11-27

**Authors:** Jinhao Li, Jiasheng Liu, Bingliang Cai, Chuansheng Yang

**Affiliations:** 1 Department of Breast, Thyroid and Head-Neck Surgery, Yuebei People’s Hospital, Shantou University Medical College, Shaoguan, Guangdong 512026 China; 2 Department of Urology, Yuebei People’s Hospital, Shantou University Medical College, Shaoguan, Guangdong 512026 China; 3 Yuebei People’s Hospital, Shantou University Medical College, No. 133, Huimin South Road, Wujiang District, Shaoguan, Guangdong 512026 China

**Keywords:** Breast cancer, eGDR, Insulin resistance, Mortality, NHANES, Competing risks

## Abstract

**Background:**

Insulin resistance (IR) is increasingly linked with poor cancer prognosis, but its impact on mortality among breast cancer patients—particularly regarding different causes of death—remains incompletely understood. The estimated glucose disposal rate (eGDR) is a surrogate index of insulin sensitivity. This study intended to investigate the links of eGDR with all-cause mortality (ACM) and breast cancer-specific mortality (BCSM) in US breast cancer survivors.

**Methods:**

Data were sourced from the 2007–2016 National Health and Nutrition Examination Survey(NHANES). A cross-sectional design was used to assess the link between eGDR and breast cancer prevalence, while a cohort design was adopted for mortality follow-up. Additionally, weighted logistic regression was leveraged to estimate the link between eGDR and breast cancer prevalence. Cox models were employed to evaluate the relationship between eGDR and ACM. Restricted cubic splines were utilized to model nonlinear links, and competing risk models were adopted to analyze BCSM.

**Results:**

Among 323 women with breast cancer, 73 deaths occurred during follow-up (median 6.2 years). After multiple confounders were controlled, a binary analysis using optimal cutoff values confirmed that higher eGDR was independently linked with lower ACM risk (HR: 0.40; 95% CI: 0.18–0.89; *P* = 0.025). This association was nonlinear, and the protective effect remained across multiple subgroups. In competing risk analysis, a potential association was observed between higher eGDR and lower BCSM risk (HR: 0.13; 95% CI: 0.02–0.99; *P* = 0.048), with no marked association observed for cardiovascular mortality.

**Conclusion:**

In US breast cancer survivors, higher eGDR was independently linked with lower ACM risk. This association may be partly driven by a reduction in BCSM, but this specific finding requires validation in larger studies due to considerable statistical uncertainty.

**Supplementary Information:**

The online version contains supplementary material available at 10.1186/s12885-025-15340-0.

## Introduction

Breast cancer, the predominant malignancy in women globally, ranks among the major causes of cancer-related deaths in women. Roughly 2.3 million new cases occur annually, constituting nearly 1/4 of all female cancer cases and 1/6 of all female cancer deaths [1, 2]. It remains a major cause of premature death in women, imposing a heavy burden on women worldwide and placing significant economic pressure on public health systems [3]. Therefore, it is crucial to investigate factors influencing breast cancer prevalence and mortality rates among women and develop effective public health interventions to reduce the disease burden.

Among these factors, insulin resistance (IR), a metabolic disorder, features diminished sensitivity to insulin, impairing an individual’s ability to take up and utilize glucose [4]. It is becoming a critical issue among breast cancer patients. Pathological types HR+/Her2- account for 65%-75% of all breast cancer cases [5], indicating that the majority of patients require standard endocrine therapy. Most chemotherapy drugs, particularly endocrine agents, have been shown to induce adverse metabolic sequelae [6] that promote IR. Concurrently, IR can accelerate breast cancer progression through crosstalk between hyperinsulinemia and key breast cancer targets, such as Her2 and ER [7–9].

Although it is known that IR is associated with an increased risk of breast cancer and poorer prognosis, evaluating it in large population studies presents challenges. The gold standard for estimating insulin sensitivity is the hyperinsulinemic euglycemic clamp technique [10]. Nonetheless, its invasion and high cost significantly restrain its utility in clinical settings and large-scale epidemiological studies [11, 12]. To this end, the estimated glucose disposal rate (eGDR) is developed and validated as a reliable, non-invasive surrogate index. The eGDR is calculated based on conventional clinical indexes, covering hypertension, waist circumference (WC), and glycated hemoglobin (HbA1c). Its validity has been demonstrated in a prospective study examining IR, diabetic nephropathy, and all-cause mortality (ACM) in diabetic populations, where lower eGDR levels are correlated with more pronounced IR [13]. Given its convenience and reliability, eGDR has become an ideal tool for assessing IR in large-scale epidemiological studies [14].

To date, numerous epidemiological analyses have observed that IR is pivotal in cancer development and patient prognosis, yet they cannot conclusively reveal causality between IR and cancer [15]. When assessing outcomes in tumor survivors, ACM is considered a more robust clinical endpoint than five-year survival rates, which are susceptible to screening and follow-up effects [16]. This study is the first to systematically investigate the association between eGDR and breast cancer prevalence in a nationally representative U.S. population, and to explore its association with ACM among breast cancer survivors in this cohort. Given this, this study intended to investigate the link between eGDR and breast cancer using the National Health and Nutrition Examination Survey (NHANES) database. We first determined the association between eGDR and breast cancer prevalence to explore its role as a risk factor. Subsequently, as the primary focus of this study, we centered on women with a history of breast cancer. Competing risks models and restricted cubic splines (RCS) were adopted to analyze the dose-response link and nonlinear characteristics between eGDR and ACM and breast cancer-specific mortality (BCSM). This study aimed to test the following hypotheses: lower eGDR is associated with a higher incidence of breast cancer in adult women; and among female breast cancer survivors, it is associated with a higher risk of ACM. The findings hold promise for identifying high-risk breast cancer survivors and providing critical information to guide targeted metabolic interventions, ultimately improving long-term survival outcomes for patients.

## Methods

### Database and study subjects

**Fig. 1:**
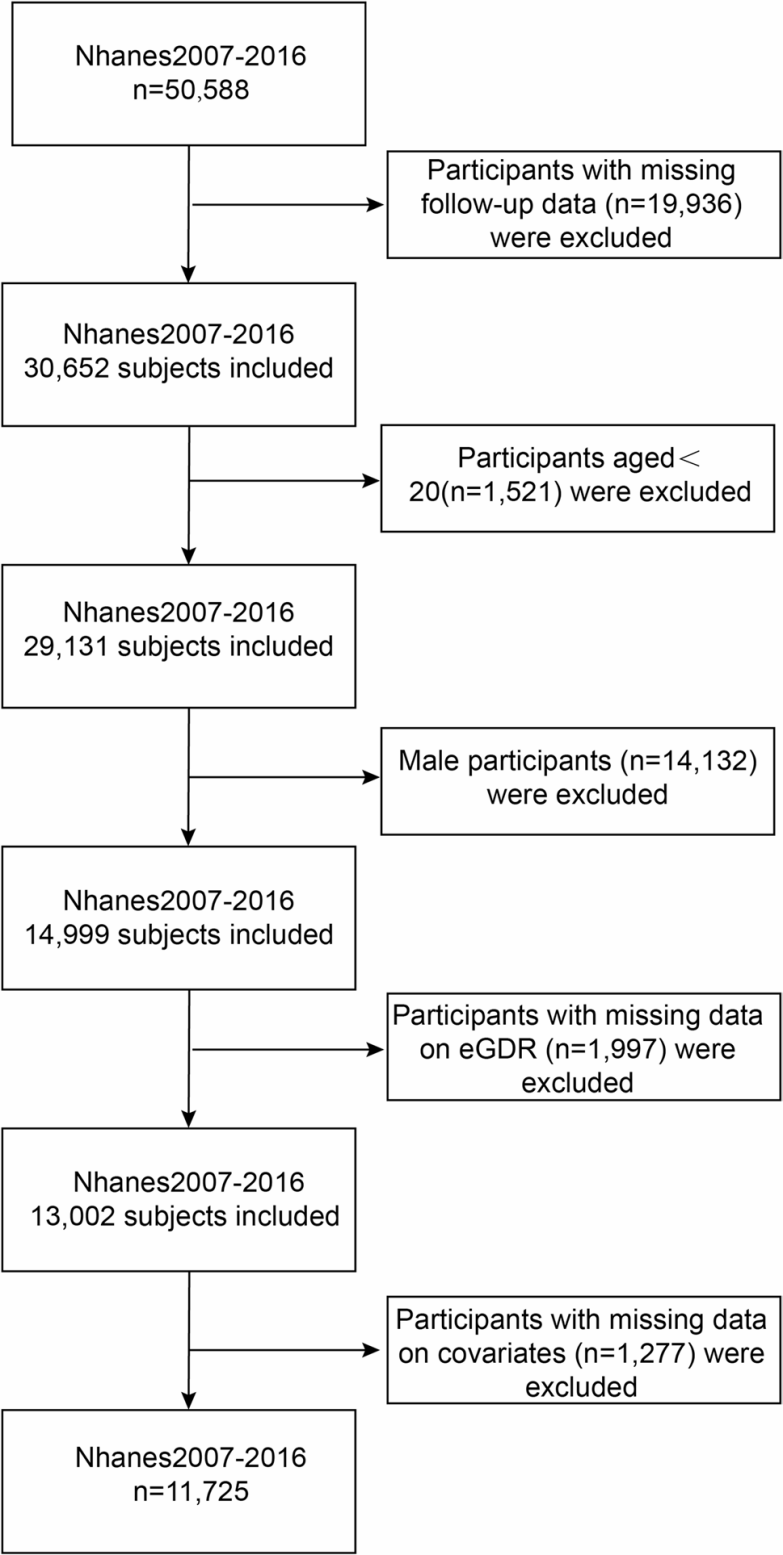
Flow chart of the study participants

This paper extracted data from the NHANES database spanning 2007–2016. The data are public in the NHANES database. The NANES database employs a stratified multistage sampling method to select representative individuals from the U.S. population, conducting a nationwide survey every two years to assess personal health and nutritional status [17]. The NHANES study protocol was ratified by the National Center for Health Statistics (NCHS) Institutional Committee, and all populations offered written informed consent. Because NHANES data are de-identified and publicly available, additional ethical approval was not required. Data from 50,588 examinees registered at NHANES mobile examination centers between 2007 and 2016 were utilized. Among them, 429 breast cancer patients were diagnosed through self-reported questionnaires. Exclusion criteria for this dataset comprised: (1) missing follow-up data (*n* = 19,936); (2) age under 20 years (*n* = 1,521); (3) male participants (*n* = 14,132); (4) absence of eGDR data (*n* = 1,997); (5) missing categorical covariates (*n* = 1,277). 11,725 participants were ultimately enrolled, including 323 breast cancer patients. Figure 1 summarizes participant selection. (Flow chart of the selection process for breast cancer survivors is shown in Supplementary Materials)

### Exposure variable

The primary exposure variable was eGDR. As mentioned in the introduction, eGDR is a clinically validated surrogate marker for IR. Its calculation formula was: 21.158 –(0.09*WC [cm])–(3.407* hypertension status[1 = Yes, 0 = No])–(0.551*HbA1c) [WC (cm), HT (yes = 1/no = 0), and HbA1c = HbA1c (%)] [18]. This subtractive formula can yield negative results for individuals with severe metabolic abnormalities. As lower eGDR is known to be associated with more severe insulin resistance, a negative value is logically interpreted as indicating a profound state of insulin resistance within the context of this model. In the following analyses, the optimal cutoff value for eGDR was determined using X-title software, classifying participants into high and low eGDR groups.

### Study outcomes

The primary endpoint for our longitudinal analysis was ACM in breast cancer survivors. The secondary outcome was breast cancer prevalence. To determine the mortality status, the NHANES Public Use Mortality File until December 31, 2019, was utilized. It was linked to the National Death Index via a probability-based matching algorithm. Death causes were specified via the ICD-10 codes. Cardiovascular disease (CVD)-related deaths were defined with ICD-10 codes as deaths from heart disease (I00-I09, I11, I13, and I20-I51) or cerebrovascular disease (I60-I69); cancer-related deaths were determined with ICD-10 codes (C50). Breast cancer survivors were defined according to diagnostic criteria cited in the literature: participants reporting a prior diagnosis of breast cancer were identified as breast cancer positive, those answering “no” were breast cancer negative, while participants with many cancer diagnoses or answering “refuse” or “don’t know’ to the breast cancer diagnosis question were excluded [19].

### Covariates

Standardized questionnaires were adopted to collect participants’ demographics, including age (years), marital status (married or cohabiting with partner, single), race/ethnicity background (White, Black, Hispanic, and other), educational attainment (below high school, high school, post- high school education), alcohol use (yes, no), poverty income ratio (PIR), and usage of estrogen medication. Body mass index (BMI, kg/m^2^) and WC were measured by experienced clinicians following standardized procedures. Clinical biochemical markers were examined through standardized protocols, including alanine aminotransferase (ALT), aspartate aminotransferase (AST), blood urea nitrogen (BUN), serum creatinine (Scr), high-density lipoprotein (HDL), low-density lipoprotein (LDL), and triglycerides (TG).

Several key categorical covariates were defined according to established guidelines or previous research criteria. Smoking status was categorized as ever smoker (defined as having smoked at least 100 cigarettes in their lifetime) versus never smoker, no matter they had quit smoking at the interview [20]. A current drinker was recognized as one who consumed over 12 alcoholic drinks in the last year [21]. CVD was identified according to self-reported diagnoses of coronary heart disease, congestive heart failure, stroke, myocardial infarction, or angina pectoris, as verified by healthcare professionals [22]. Depressive status was estimated via the Patient Health Questionnaire-9 (PHQ-9), which assessed depression severity in cancer survivors 2 weeks before. Its validity and performance have been verified in cancer patients [23]. In this study, individuals with ≥ 10 scores were defined as being in a depressive state [24]. (Details are provided in Supplementary Material-Description of Covariates)

According to the 2017 ACCA Guideline Standard [25], the participant was diagnosed with hypertension if one of the criteria was met: (1) mean systolic blood pressure ≥ 130 mmHg; (2) mean diastolic blood pressure ≥ 80 mmHg; (3) prior diagnosis of hypertension; (4) current administration of antihypertensive medication. Diabetes was determined as follows: (1) previously diagnosed diabetes (currently using insulin or oral hypoglycemic drugs); (2) HbA1c ≥ 6.5%; (3) fasting blood glucose ≥ 126 mg/dL (7.0 mmol/L) or 2-hour glucose ≥ 200 mg/dL (11.1 mmol/L) during an oral glucose tolerance test; or (4) random blood glucose ≥ 200 mg/dL (11.1 mmol/L) [22].

Missing data in covariates were handled as follows: continuous covariates with a missing rate > 20% were excluded; for continuous covariates with a missing rate ≤ 20%, missing values were imputed via the random forest algorithm. Specifically, the variables that required imputation were ALT (1.7% missing), AST (1.7% missing), Blood urea nitrogen (1.7% missing), Serum Creatinine (1.7% missing), TG (1.7% missing), BMI (0.2% missing), family PIR (8.3% missing), and LDL (1.2% missing). A detailed summary of missing data for all covariates is provided in Supplementary Table 1.

### Statistical analysis

This study followed the CDC’s complex sampling design guidelines for NHANES data analysis, incorporating appropriate weights in all analyses. Based on the quartile distribution of eGDR (Q1 was the lowest quartile and Q4 was the highest quartile), all subjects were assigned to four subgroups. Breast cancer survivors were further classified into two subgroups according to their survival status. Following normality testing, all continuous variables in this study were confirmed as non-normally distributed, depicted as medians and interquartile ranges, and analyzed via ANOVA or Kruskal-Wallis tests. Categorical variables were delineated as counts (weighted percentages), and comparisons were performed utilizing weighted chi-square tests. A weighted restricted cubic spline (RCS) model was employed for analysis, with three knots positioned at the 10th, 50th, and 90th percentiles of the eGDR distribution. Based on the shape of the RCS curve and nonlinearity test results, the Xtitle software determined the optimal cutoff value for eGDR to be 8.8. Survivors were stratified into high eGDR (≥ 8.8) and low eGDR (< 8.8) groups according to this cutoff. Survival curves were plotted via the Kaplan-Meier method, and the log-rank test was employed to judge differences in ACM. Before constructing the multivariate model, multicollinearity among covariates was judged by calculating the variance inflation factor (VIF). All variables showed VIF values < 5, implying no marked multicollinearity issues. Notably, the individual components of the eGDR formula (waist circumference, hypertension status, and HbA1c) were not considered for inclusion as covariates in the adjusted models to prevent over-adjustment and inherent multicollinearity with the primary exposure variable. Weighted multifactor logistic regression and weighted multifactor Cox models were constructed. Logistic regression was leveraged to ascertain the link between eGDR and breast cancer prevalence in all female participants. Four progressively adjusted models were constructed: Model 1, unadjusted; Model 2, only controlled age; Model 3 controlled age, race, education level, and PIR; Model 4 considered age, race, marital status, PIR, BMI, history of diabetes, smoking and drinking status, and CVD status, shown as odds ratios (OR) and 95% confidence intervals (95% CI). Cox regression was leveraged to ascertain the link between eGDR and ACM in breast cancer survivors, and three progressively adjusted models were constructed: Model 1, unadjusted; Model 2, controlled age, race, education level, and PIR; Model 3, considered age, race, marital status, PIR, BMI, history of diabetes, smoking and drinking status, and CVD status, with results manifested as hazard ratios (HR) and 95% CI. A competing risk model was constructed to assess the cumulative probability of CVD death and BCSM across different eGDR groups by plotting cumulative incidence function (CIF) curves. Gray’s tests were employed to compare intergroup differences. Cause-specific Cox models were established to calculate cause-specific HRs, further analyzing competing risks for mortality outcomes [26]. Sensitivity and subgroup analyses were performed to further prove the robustness and credibility of the results [27]. All data analyses were performed in R software V4.4.2. All tests were two-tailed, with *P* < 0.05 implying statistically significant.

## Results

### Baseline traits

11,725 participants were enrolled. Data were allocated into four subgroups based on eGDR quartiles. Baseline characteristics for all participants are outlined in Table 1. The median eGDR was 7.528, with interquartile ranges of -3.98 to 5.37, 5.37 to 7.53, 7.53 to 10.04, and 10.04 to 13.01. The median age of included participants was 48 years. The overall breast cancer prevalence was 2.7%, and the ACM proportion was 6.3%. Compared to the group with lower eGDR, those with higher eGDR were younger, had higher educational attainment, tended to be non-Hispanic whites, had better household economic status, had lower proportions of smokers and drinkers, were more likely to engage in daily moderate physical activity, had lower prevalence of hypertension, diabetes, and CVD, and exhibited lower rates of estrogen medication use. Participants with higher eGDR exhibited lower Scr, TG, BMI, WC, HbA1c, and LDL, and higher HDL (*P* < 0.001), with no significant difference in BUN. Furthermore, as eGDR increased, the prevalence of breast cancer, ACM, and CVD all significantly decreased.

**Table 1:** Basic characteristics of participants from NHANES 2007–2016 grouped by eGDR quartiles

Variables	Overall *N* = 97,007,971^*2*^	eGDR-Q1 *N* = 20,807,774^*2*^ (-3.98-5.37)	eGDR-Q2 *N* = 23,186,015^*2*^ (5.37–7.53)	eGDR-Q3 *N* = 25,071,503^*2*^ (7.53–10.04)	eGDR-Q4 *N* = 27,942,679^*2*^ (10.04–13.01)	*P*-Value
Age, (years)	48(34.00,61.00)	58(47.00,67.00)	57(45.00,69.00)	44(32.00,56.00)	36(26.00,48.00)	< 0.001
Marital_status, n(%)						< 0.001
Living alone	5,337(39.30%)	1,506(43.34%)	1,388(41.07%)	1,189(35.40%)	1,254(38.33%)	
Married or living with partner	6,388(60.70%)	1,425(56.66%)	1,543(58.93%)	1,741(64.60%)	1,679(61.67%)	
Education_levels, n(%)						< 0.001
Less than high school	2,797(15.40%)	891(20.61%)	773(17.38%)	695(15.39%)	438(9.91%)	
High school or equivalent	2,529(21.03%)	700(24.93%)	714(23.88%)	629(21.21%)	486(15.61%)	
Above high school	6,399(63.56%)	1,340(54.47%)	1,444(58.75%)	1,606(63.39%)	2,009(74.48%)	
Race, n (%)						< 0.001
Mexican American	1,842(7.82%)	466(7.42%)	406(6.48%)	586(10.42%)	384(6.89%)	
Other Hispanic	1,374(5.59%)	310(4.42%)	334(4.73%)	368(6.54%)	362(6.34%)	
Non-Hispanic White	5,005(68.52%)	1,156(65.88%)	1,323(71.47%)	1,204(66.68%)	1,322(69.70%)	
Non-Hispanic Black	2,371(11.19%)	862(17.82%)	595(11.11%)	529(10.22%)	385(7.20%)	
Other Race - Including Multi-Racial	1,133(6.87%)	137(4.47%)	273(6.21%)	243(6.14%)	480(9.87%)	
Drinking status, n(%)						< 0.001
No	4,576(30.78%)	1,358(39.27%)	1,234(33.29%)	1,077(29.60%)	907(23.42%)	
Yes	7,149(69.22%)	1,573(60.73%)	1,697(66.71%)	1,853(70.40%)	2,026(76.58%)	
Smoking status, n(%)						< 0.001
No	7,480(61.00%)	1,724(55.89%)	1,834(58.63%)	1,852(60.39%)	2,070(67.33%)	
Yes	4,245(39.00%)	1,207(44.11%)	1,097(41.37%)	1,078(39.61%)	863(32.67%)	
Vigorous work acticity, n(%)						< 0.001
No	9,715(78.73%)	2,749(92.79%)	2,587(85.97%)	2,383(78.37%)	1,996(62.60%)	
Yes	2,010(21.27%)	182(7.21%)	344(14.03%)	547(21.63%)	937(37.40%)	
Moderate work acticity, n(%)						< 0.001
No	6,966(53.47%)	2,035(67.16%)	1,777(54.43%)	1,645(50.28%)	1,509(45.33%)	
Yes	4,759(46.53%)	896(32.84%)	1,154(45.57%)	1,285(49.72%)	1,424(54.67%)	
Hypertension, n(%)						< 0.001
No	6,009(55.37%)	81(2.57%)	442(14.99%)	2,553(86.79%)	2,933(100.00%)	
Yes	5,716(44.63%)	2,850(97.43%)	2,489(85.01%)	377(13.21%)	0(0.00%)	
Diabetes, n(%)						< 0.001
No	9,669(86.83%)	1,623(61.48%)	2,460(88.11%)	2,696(93.26%)	2,890(98.88%)	
Yes	2,056(13.17%)	1,308(38.52%)	471(11.89%)	234(6.74%)	43(1.12%)	
CVD, n(%)						< 0.001
No	10,714(92.85%)	2,430(84.53%)	2,600(90.08%)	2,806(96.25%)	2,878(98.28%)	
Yes	1,011(7.15%)	501(15.47%)	331(9.92%)	124(3.75%)	55(1.72%)	
Depression, n(%)						< 0.001
No	10,745(92.93%)	2,564(88.72%)	2,703(93.75%)	2,690(92.20%)	2,788(96.04%)	
Yes	980(7.07%)	367(11.28%)	228(6.25%)	240(7.80%)	145(3.96%)	
Estrogen use, n(%)						< 0.001
No	9,518(78.29%)	2,231(71.30%)	2,133(69.05%)	2,498(81.87%)	2,656(87.94%)	
Yes	2,207(21.71%)	700(28.70%)	798(30.95%)	432(18.13%)	277(12.06%)	
ALT, U/L	18(15.00,23.00)	21(16.00,27.00)	19(16.00,24.00)	18(15.00,23.00)	16(14.00,20.00)	< 0.001
AST, U/L	22(19.00,26.00)	22(19.00,27.00)	23(19.00,27.00)	21(18.00,25.00)	21(18.00,24.00)	< 0.001
Blood urea nitrogen, (mg/dL)	12(9.00,15.00)	13(10.00,17.00)	13(10.00,16.00)	11(9.00,14.00)	11(9.00,13.00)	< 0.001
Serum Creatinine (mg/dL)	0.74(0.65,0.84)	0.78(0.68,0.90)	0.76(0.67,0.87)	0.73(0.64,0.82)	0.72(0.65,0.81)	< 0.001
Triglycerides (mg/dL)	113(76.00,170.00)	155(105.00,220.00)	126(87.00,183.00)	116(79.00,169.00)	80(58.00,113.00)	< 0.001
BMI, (kg/m^2^)	27.70(23.70,33.20)	35.70(32.00,40.69)	27.50(25.00,31.00)	29.27(26.10,32.76)	22.90(21.00,24.91)	< 0.001
Waist Circumference (cm)	95.00(84.50,107.10)	113.60(107.20,123.10)	94.90(88.80,100.40)	98.40(93.00,105.60)	81.70(76.50,86.30)	< 0.001
Family PIR	2.77(1.45,4.80)	2.26(1.26,4.06)	2.74(1.49,4.71)	2.68(1.37,4.64)	3.43(1.71,5.00)	< 0.001
HbA1c,%	5.40(5.20,5.80)	5.90(5.50,6.50)	5.50(5.30,5.80)	5.40(5.20,5.70)	5.20(5.00,5.40)	< 0.001
HDL, (mg/dL)	57(47.00,68.00)	49(42.00,58.00)	57(47.00,69.00)	55(46.00,66.00)	63(53.00,74.00)	< 0.001
LDL, (mg/dL)	195(169.00,222.00)	196(170.00,224.00)	202(177.00,231.00)	198(171.00,225.00)	184(161.00,210.00)	< 0.001
All-cause mortality, n(%)	920(6.30%)	358(10.48%)	347(10.25%)	136(3.82%)	79(2.14%)	< 0.001
Breast cancer, n(%)						< 0.001
No	11,402(97.31%)	2,821(96.46%)	2,817(95.82%)	2,867(97.67%)	2,897(98.87%)	
Yes	323(2.69%)	110(3.54%)	114(4.18%)	63(2.33%)	36(1.13%)	

*Continuous data were presented median (25th–75th percentile), category data were presented as the proportion of unweighted sample size (percentage)

*Abbreviations: *ALT* Alanine Aminotransferase, *AST* Aspartate Aminotransferase, *BMI* Body Mass Index, *BUN* Blood Urea Nitrogen, *CVD* Cardiovascular Disease, *eGDR* Estimated Glucose Disposal Rate, *HbA1c* Glycated Hemoglobin, *HDL* High-Density Lipoprotein, *LDL* Low-Density Lipoprotein, *NHANES* National Health and Nutrition Examination Survey, *PIR* Poverty-to-Income Ratio, *Q1-Q4* Quartile 1 to Quartile 4, *Scr* Serum Creatinine

***P value < 0.001, **P value < 0.01, *P value < 0.05

^*1*^ Total weighted sample size (N)

^*2*^ Median(Q1,Q3); n (unweighted)(%)

^*3*^ Design-based KruskalWallis test; Pearson’s X^2: Rao & Scott adjustment

Participants with a prior diagnosis of breast cancer (*n* = 323) were grouped according to their mortality status during follow-up. Table 2 details their baseline characteristics. 73 deaths (22.6%) occurred. Compared with survivors, participants in the death group were markedly older (median age: 80 vs. 65 years, *P* < 0.001), had reduced educational level (*P* = 0.007), and bore a heavier burden of comorbidities, including hypertension (*P* = 0.006) and CVD (*P* = 0.013). Baseline eGDR was also substantially lower in the death group (median: 6.15 vs. 6.44, *P* = 0.031).

**Table 2:** Baseline characteristics of breast cancer participants from NHANES 2007–2016 stratified by All-Cause mortality status

Variables	Overall *N* = 2,606,088^*2*^	Survival *N* = 2,085,300^*2*^	Death *N* = 520,788^*2*^	*P*-Value^3^
Age, (years)	67(59.00,77.00)	65(57.00,74.00)	80(72.00,80.00)	< 0.001
Marital_status, n(%)				0.105
Living alone	156(44.02%)	115(41.44%)	41(54.37%)	
Married or living with partner	167(55.98%)	135(58.56%)	32(45.63%)	
Education_levels, n(%)				0.007
Less than high school	68(12.40%)	47(9.93%)	21(22.33%)	
High school or equivalent	72(22.76%)	54(21.65%)	18(27.17%)	
Above high school	183(64.84%)	149(68.42%)	34(50.50%)	
Race, n (%)				0.513
Mexican American	29(2.65%)	27(3.06%)	2(1.01%)	
Other Hispanic	31(3.92%)	29(4.48%)	2(1.67%)	
Non-Hispanic White	191(81.22%)	137(80.34%)	54(84.75%)	
Non-Hispanic Black	50(7.58%)	40(7.70%)	10(7.12%)	
Other Race - Including Multi-Racial	22(4.62%)	17(4.41%)	5(5.46%)	
Drinking status, n(%)				0.073
No	140(37.25%)	102(34.27%)	38(49.17%)	
Yes	183(62.75%)	148(65.73%)	35(50.83%)	
Smoking status, n(%)				0.112
No	192(58.57%)	154(61.24%)	38(47.91%)	
Yes	131(41.43%)	96(38.76%)	35(52.09%)	
Vigorous work acticity, n(%)				0.004
No	295(87.44%)	224(84.87%)	71(97.72%)	
Yes	28(12.56%)	26(15.13%)	2(2.28%)	
Moderate work acticity, n(%)				0.185
No	200(57.22%)	150(55.18%)	50(65.39%)	
Yes	123(42.78%)	100(44.82%)	23(34.61%)	
Hypertension, n(%)				0.006
No	96(34.08%)	84(38.37%)	12(16.91%)	
Yes	227(65.92%)	166(61.63%)	61(83.09%)	
Diabetes, n(%)				0.101
No	224(73.99%)	176(75.89%)	48(66.40%)	
Yes	99(26.01%)	74(24.11%)	25(33.60%)	
CVD, n(%)				0.013
No	262(82.13%)	211(85.19%)	51(69.91%)	
Yes	61(17.87%)	39(14.81%)	22(30.09%)	
Depression, n(%)				0.738
No	295(93.90%)	228(93.67%)	67(94.79%)	
Yes	28(6.10%)	22(6.33%)	6(5.21%)	
Estrogen use, n(%)				0.601
No	222(66.66%)	173(65.77%)	49(70.23%)	
Yes	101(33.34%)	77(34.23%)	24(29.77%)	
ALT, U/L	19(16.00,24.00)	20(16.00,25.00)	17(15.00,21.00)	0.002
AST, U/L	23(20.00,27.00)	23(20.00,27.00)	22(19.00,26.00)	0.103
Blood urea nitrogen, (mg/dL)	14.0(11.00,18.00)	14.0(11.00,17.00)	16.0(12.00,22.00)	< 0.001
Serum Creatinine (mg/dL)	0.79(0.70,0.94)	0.78(0.69,0.91)	0.90(0.72,1.05)	0.005
Triglycerides (mg/dL)	136(93.00,197.00)	138(92.00,197.00)	129(101.00,192.00)	0.862
BMI, kg/m^2^	28.15(24.18,33.13)	28.20(24.40,33.50)	27.44(23.18,31.13)	0.234
Waist Circumference (cm)	97.80(88.20,107.40)	98.00(88.20,107.30)	97.50(89.00,107.40)	0.644
Family PIR	2.90(1.76,5.00)	3.14(1.85,5.00)	1.89(1.52,4.25)	0.023
HbA1c,%	5.70(5.40,6.00)	5.70(5.40,6.00)	5.80(5.50,6.10)	0.112
HDL, (mg/dL)	59(48.00,70.00)	59(48.00,69.00)	58(48.00,74.00)	0.865
LDL, (mg/dL)	201(174.00,227.00)	202(174.00,225.00)	196(175.00,239.00)	0.840
eGDR	6.34(5.26,8.55)	6.44(5.26,8.86)	6.15(5.15,7.39)	0.031

*Continuous data were presented median (25th–75th percentile), category data were presented as the proportion of unweighted sample size (percentage)

*Abbreviations: *ALT* Alanine Aminotransferase, *AST* Aspartate Aminotransferase, *BMI* Body Mass Index, *BUN* Blood Urea Nitrogen, *CVD* Cardiovascular Disease, *eGDR* Estimated Glucose Disposal Rate, *HbA1c* Glycated Hemoglobin, *HDL* High-Density Lipoprotein, *LDL* Low-Density Lipoprotein, *NHANES* National Health and Nutrition Examination Survey, *PIR* Poverty-to-Income Ratio, *Q1-Q4* Quartile 1 to Quartile 4, *Scr* Serum Creatinine

***P value < 0.001, **P value < 0.01, *P value < 0.05

^*1*^Total weighted sample size (N)

^*2*^ Median(Q1,Q3); n (unweighted)(%)

^*3*^ Design-based KruskalWallis test; Pearson’s X^2: Rao & Scott adjustment

### Relationship between eGDR and breast cancer prevalence

Among 11,725 female participants in the unadjusted model, higher eGDR was notably linked with a lower breast cancer prevalence (OR = 0.88, 95% CI: 0.84–0.91, *P* < 0.001). When analyzed by quartile grouping, Q3 and Q4 groups exhibited a significantly reduced breast cancer risk relative to the lowest Q1 group (Q3: OR = 0.650, 95% CI: 0.84–0.91, *P* = 0.044; Q4: OR = 0.312, 95% CI: 0.20–0.39, *P* < 0.001). However, this association was substantially attenuated and lost statistical significance after adjusting for age alone (Model 2: OR = 0.999, 95% CI: 0.94–1.05, *P* = 0.96) (Table 3).

**Table 3:** Breast cancer prevalence according to eGDR among female in NHANES 2007–2016

Characteristic	Model 1			Model 2			Model 3			Model 4		
	OR	95%CI	*P*-value	OR	95%CI	*P*-value	OR	95%CI	*P*-value	OR	95%CI	*P*-value
Breast cancer prevalence	0.876	0.84,0.91	<0.001	0.999	0.95, 1.05	0.96	0.992	0.94,1.04	0.752	0.997	0.91,1.09	0.938
eGDR quartiles												
Q1	Ref			Ref			Ref			Ref		
Q2	1.189	0.84,1.69	0.328	1.241	0.87, 1.77	0.22	1.207	0.84,1.74	0.311	1.270	0.79,2.04	0.320
Q3	0.650	0.43,0.99	0.044	1.169	0.78, 1.76	0.45	1.131	0.75,1.71	0.552	1.246	0.74,2.08	0.397
Q4	0.312	0.20,0.49	<0.001	0.871	0.56, 1.36	0.54	0.815	0.51,1.30	0.383	0.868	0.46,1.64	0.659

OR odds ratio, 95% CI 95% confidence interval

Model 1: unadjusted

Model 2: adjusted for age

Model 3: adjusted for age, race, education level, the family poverty income ratio

Model 4: adjusted for age, race, education level, marital status, family poverty income ratio, Body Mass Index, diabetes, alcohol use, smoked, CVD

### RCS analysis

**Fig. 2:**
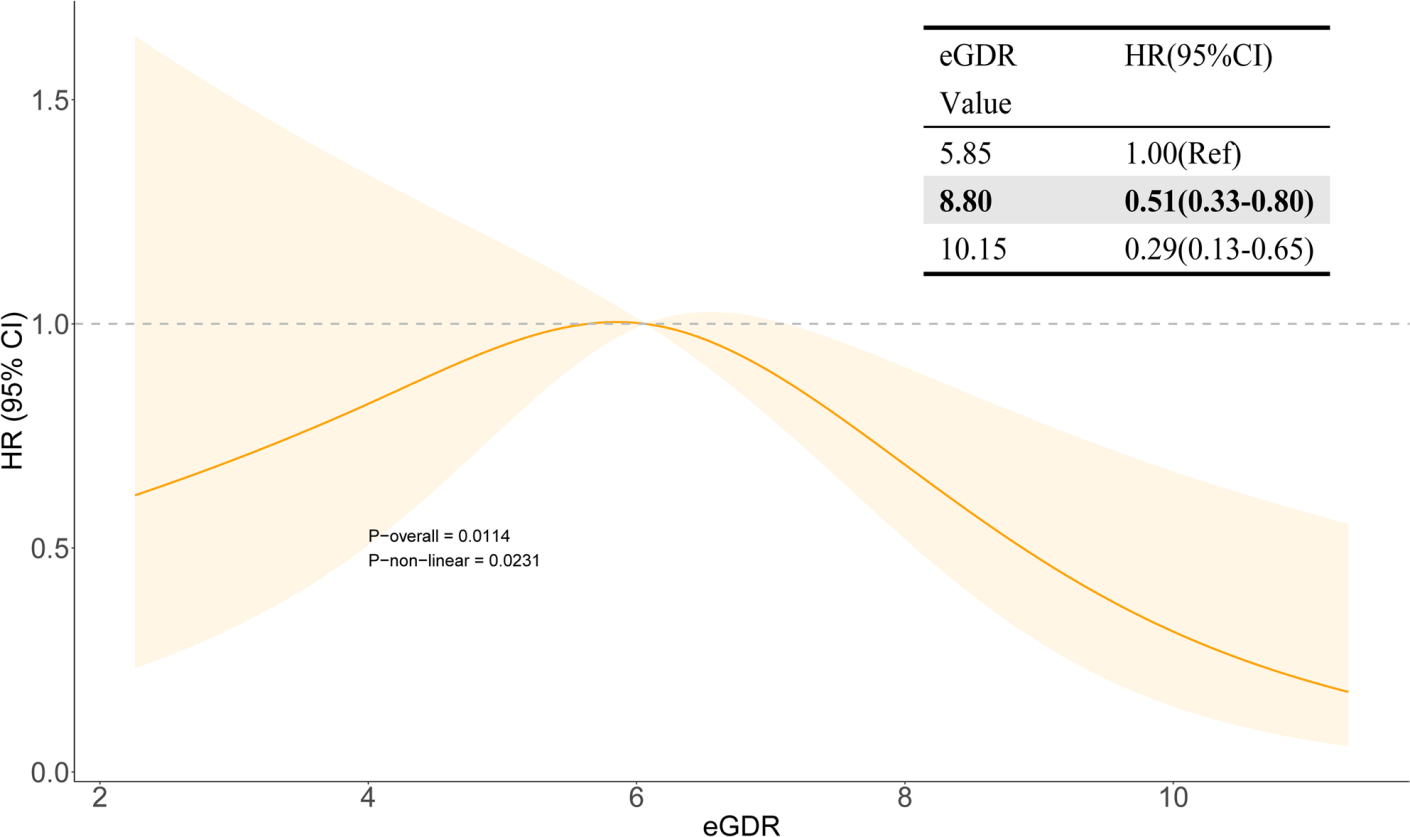
RCS analysis between eGDR and all-cause mortality in breast cancer patients. The inset table provides the specific HRs and 95% CIs at key data-driven eGDR values. *Abbreviations: CI, confidence interval; eGDR, estimated glucose disposal rate; HR, hazard ratio

RCS curves (Fig. 2) revealed a nonlinear link between eGDR and ACM in breast cancer survivors (P-non-linear = 0.0231). Using the risk peak point (HR = 1, eGDR = 5.85) as reference, the protective effect was statistically significant when eGDR ≥ 8.80 (HR = 0.51, 95% CI: 0.33–0.80). Furthermore, the risk decreased at the 90th percentile of eGDR distribution in the study population (eGDR = 10.15) (HR = 0.29, 95% CI: 0.13–0.65), indicating a clear dose-response relationship (Fig. 2).

### Kaplan-Meier survival analysis

**Fig. 3:**
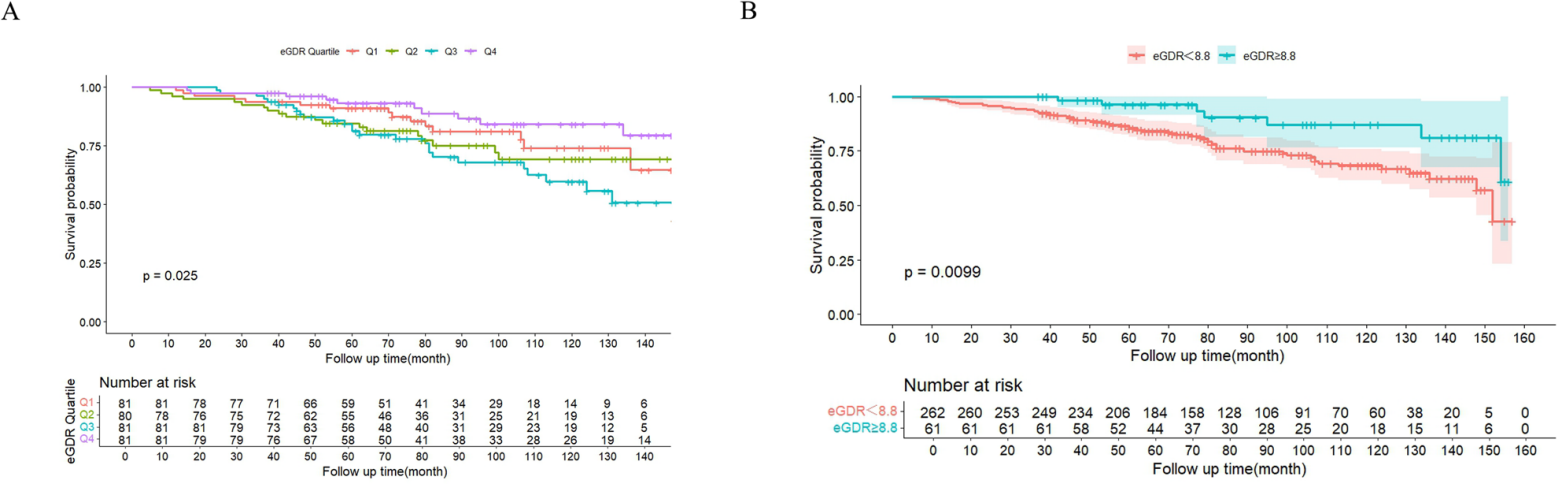
Kaplan-Meier survival analysis curves for all-cause mortality among eGDR groups. **A** eGDR quartiles **B** eGDR cutoff. The tables below the plots indicate the number of individuals at risk in each group at specific time points

Participants with higher eGDR levels exhibited considerably lower ACM risk. Survival curves across different eGDR levels demonstrated significant differences (Fig. 3).

### Relationship between eGDR and ACM

In the cohort of breast cancer survivors, weighted multivariate Cox regression model (Table 4) indicated that the high eGDR group was prominently linked with a reduced ACM risk. After adjusting for all covariates, the protective effect remained robust (HR: 0.401; 95% CI, 0.18–0.89; *P* = 0.025).

**Table 4:** Cox regression analysis of eGDR and all-cause mortality in female patients with breast cancer

Characteristic	Model 1			Model 2			Model 3		
	HR	95%CI	P-value	HR	95%CI	P-value	HR	95%CI	*P*-value
All-cause mortality	0.879	0.81,0.96	0.003	0.924	0.82,1.04	0.197	0.865	0.69,1.09	0.217
eGDR(<8.8)	Ref			Ref			Ref		
eGDR(≥ 8.8)	0.240	0.11,0.53	<0.001	0.369	0.18,0.77	0.008	0.401	0.18,0.89	0.025

OR odds ratio, 95% CI 95% confidence interval

Model 1: unadjusted

Model 2: adjusted for age, race, education level, the family poverty income ratio

Model 3: adjusted for age, race, education level, marital status, family poverty income ratio, Body Mass Index, diabetes, alcohol use, smoked, CVD

### Competing risk analysis for BCSM

Based on prior epidemiological evidence and the BCSM distribution data from the cohort of breast cancer survivors, we conducted a competing risk analysis on the two most common causes of death in this cohort, CVD mortality and BCSM mortality, which together accounted for 57.53% of all deaths [26].

**Fig. 4:**
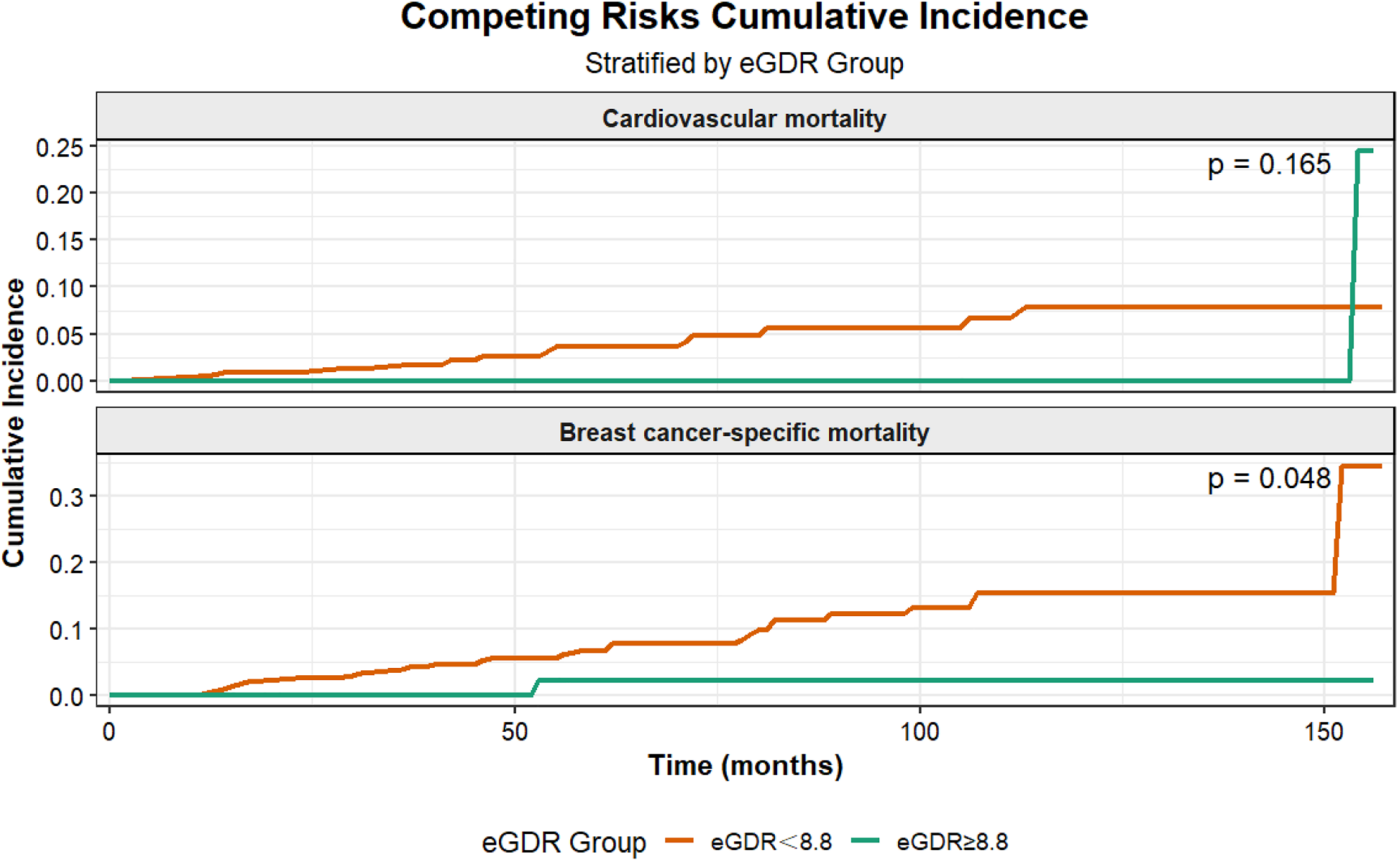
Competing risks cumulative incidence of cardiovascular and breast cancer-specific mortality. *Abbreviations: CIF, Cumulative Incidence Function; eGDR, Estimated Glucose Disposal Rate

The CIF curve is shown in Fig. 4. For CVD mortality, no significant difference was revealed in cumulative incidence between the high eGDR group (eGDR ≥ 8.8) and the low eGDR group (eGDR < 8.8) (Gray’s test, *P* = 0.165). In contrast, the high eGDR group presented a notably lower cumulative incidence of BCSM (Gray’s test, *P* = 0.048).

In the cause-specific risk models, an association between high eGDR and lower BCSM risk was observed (HR: 0.13; 95% CI: 0.02–0.99; *P* = 0.048), though the confidence interval was wide, indicating statistical imprecision. No significant association was found for CVD mortality (HR: 0.23; 95% CI: 0.03–1.85; *P* = 0.165).

### Subgroup analysis

**Fig. 5:**
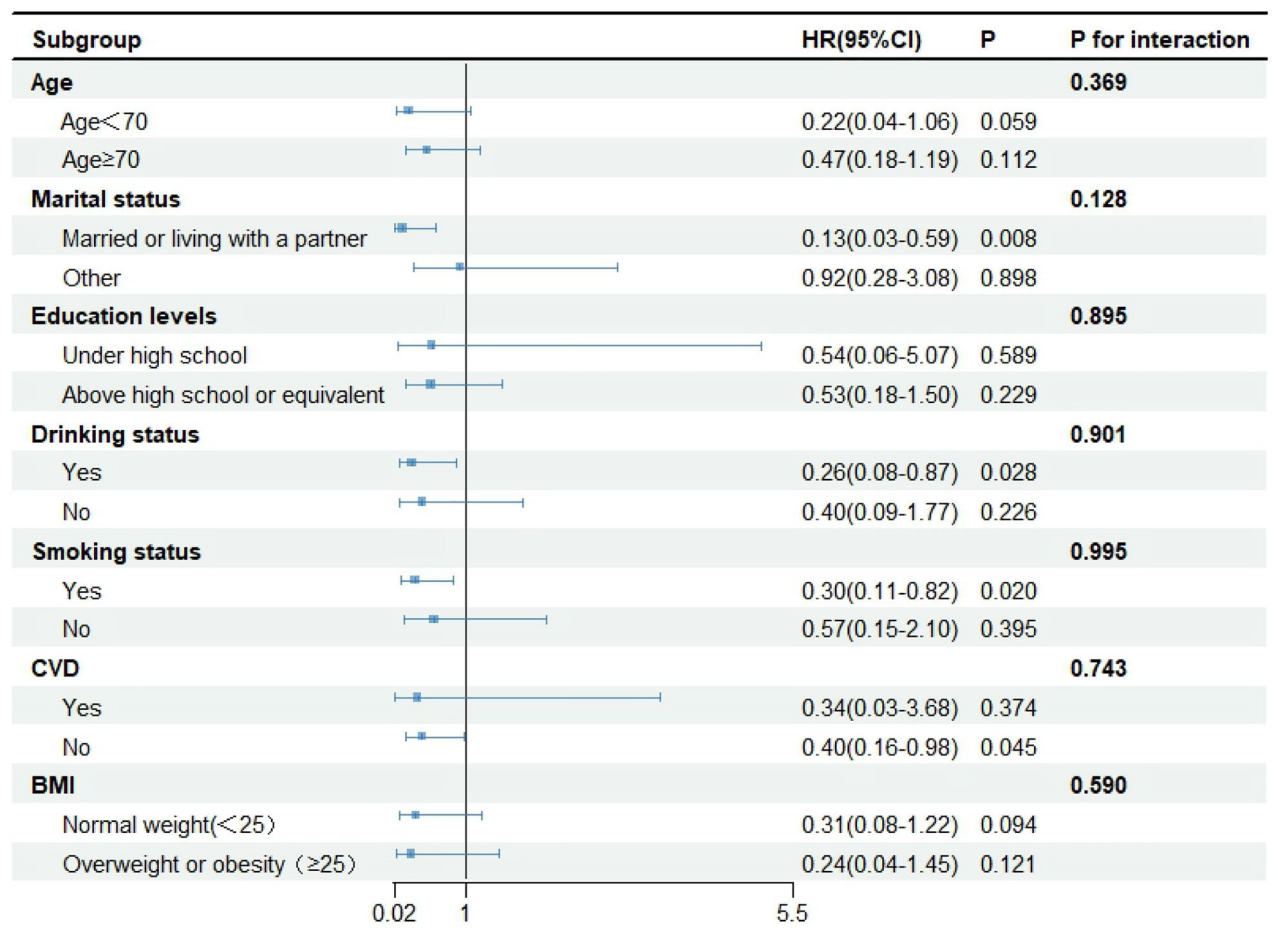
Subgroup analysis of multi-variable adjusted association of eGDR with all-cause mortality. *Abbreviations: BMI, body mass index; CI, confidence interval; CVD, cardiovascular disease; eGDR, estimated glucose disposal rate; HR, hazard ratio

To test the robustness of the association between eGDR and ACM in breast cancer survivors, subgroup analyses were performed. No pronounced interactions between eGDR and these stratification variables were revealed in any subgroup analysis (all P for interaction > 0.05) (Fig. 5).

## Discussion

This study employed data from the 2007–2016 NHANES to examine the link between eGDR and ACM from breast cancer. Among breast cancer survivors, those who ultimately died were older and had a heavier burden of comorbidities (such as hypertension and CVD) than survivors, with notable differences in eGDR (Table 2). After multivariable adjustments for these baseline differences, our study suggested an independent and significant link between higher eGDR levels and lower ACM, which also exhibited a nonlinear pattern. While our analysis suggested this association was independent of measured confounders, we acknowledge the potential for residual confounding from factors like age and comorbidities. This protective effect demonstrated consistent robustness across age, marital status, education level, smoking and drinking habits, history of CVD, and BMI. Notably, our findings suggest that this survival advantage may be primarily driven by a reduced risk of BCSM, rather than CVD mortality. If validated in future studies, these findings could provide new insights for guiding prognostic management in breast cancer survivors through improved metabolic health.

During data analysis in this study, an initial notable association between eGDR and breast cancer prevalence was observed, which was substantially attenuated and lost statistical significance after adjusting for confounders, particularly age. This strongly suggests that age is a potent confounding factor that mediates the relationship between eGDR and breast cancer prevalence. This finding aligns with previous research, which concludes that cancer risk rises exponentially with increasing age [28]. In the multivariate model, the impact of age on breast cancer prevalence far exceeds that of eGDR. Based on prior research, this confounding effect may be twofold. Aging is a strong risk indicator for cancer; the aging process itself is accompanied by metabolic decline, such as reduced insulin sensitivity and impaired glucose tolerance, both of which directly influence eGDR levels [29]. Therefore, the observed age-related attenuation of the association involves complex interactions. Future research can focus on the potential link between eGDR and breast cancer survivors at different stages.

IR is characterized by reduced insulin sensitivity, manifesting as weakened glucose utilization and metabolic abnormalities. eGDR combines indicators associated with blood glucose, blood pressure, and obesity to comprehensively assess IR [18]. Numerous reports have recognized a strong link between IR and breast cancer survivors [30–32]. Our findings further elucidated the close relationship between eGDR and ACM in breast cancer. Our findings are highly consistent with evidence from large prospective cohorts. For example, a landmark study from the Women’s Health Initiative demonstrated that elevated metabolic syndrome scores were independently associated with a 53% increase in ACM and a 44% increase in BCSM among breast cancer survivors during long-term follow-up [33]. Given that metabolic syndrome largely represents the clinical manifestation of underlying IR as measured by eGDR, this finding provides robust external support for our research outcomes. However, before delving into its potential biological mechanisms, it is imperative to acknowledge and consider alternative explanations, particularly reverse causality.Among breast cancer survivors in the progressive stage, cachexia may lead to weight loss and WC reduction, which paradoxically elevates the calculated eGDR value and may obscure the true risks of poor metabolic health.Despite this significant possibility, our findings are highly consistent with extensive biological evidence, suggesting that IR may indeed play a key pathophysiological role, partially explaining compensatory hyperinsulinemia. When tissues become insulin-resistant, cellular responsiveness to insulin diminishes. In response, pancreatic β-cells increase insulin concentration in a compensatory manner to maintain blood glucose homeostasis, leading to persistently elevated circulating insulin levels, a condition known as hyperinsulinemia [34]. Increasing evidence implicates hyperinsulinemia as a more significant tumor driver than hyperglycemia itself. Cancer cells mainly rely on aerobic glycolysis, not mitochondrial oxidative phosphorylation, for energy production owing to altered metabolism (the Warburg effect) [35]. However, this does not mean that hyperglycemia promotes tumor initiation and progression. Multiple in vivo studies indicate that hyperglycemia alone may not promote tumor progression in the absence of hyperinsulinemia [36–38]. Therefore, hyperinsulinemia induced by IR represents the pivotal mechanism driving cancer progression [36]. Furthermore, hyperinsulinemia promotes cancer progression by activating the insulin/IGF-1 axis. Insulin is a part of the insulin/IGF superfamily, which includes IGF-1, IGF-2, IGF-binding proteins, and their receptors [39]. The insulin signaling regulates and activates signaling cascade reactions such as PI3K/Akt/mTOR and Ras/MAPK, thereby controlling cell proliferation and survival [40, 41]. Hyperinsulinemia reduces hepatic synthesis of IGFBP-1 and IGFBP-2, thereby indirectly enhancing this signaling pathway and activating multiple classical oncogenic pathways, ultimately accelerating breast cancer progression [42]. Additionally, animal studies have demonstrated that exposure to high levels of IGF-1 enhances breast cancer growth and metastasis [43]. The IGF-1R pathway interacts with key therapeutic targets such as Her2 and ER, thereby influencing breast cancer treatment [7–9]. Moreover, IR frequently creates a systemic microenvironment that promotes cancer progression. This state is closely related to systemic chronic low-grade inflammation, characterized by elevated levels of cytokines, which create favorable conditions for tumor progression [44–47]. By integrating these complex biological pathways, our findings provide epidemiological support for the hypothesis that lower eGDR (i.e., IR) serves as a significant indicator of poorer prognosis in breast cancer patients. In summary, our findings, while exploratory, hold potentially significant clinical implications. However, it is crucial to interpret the findings within the scope of their limitations, such as the potential for reverse causation and the limited generalizability of our small survivor cohort. As an easily calculable index that integrates multiple routine clinical metrics, eGDR shows promise as a convenient, though preliminary, tool for assessing metabolic risk in breast cancer survivors. It must be emphasized that eGDR is currently a research tool and has not been validated for routine clinical use in oncology. Therefore, further research is required to validate eGDR as a reliable prognostic marker. If its utility is confirmed in larger, more detailed cohort studies, monitoring eGDR could help clinicians to identify survivors at high mortality risk earlier and implement more proactive interventions. Such future research could also clarify whether interventions that improve eGDR—such as early management of hyperglycemia associated with the PI3K inhibitor aprelisib [48] or combining lapatinib with metformin in survivors with lapatinib-resistant breast cancer [49] could yield survival benefits for patients.

## Limitations

This study has certain limitations: (1) Given database constraints, we were unable to obtain key clinical and pathological information regarding breast cancer, such as tumor staging (e.g., TNM staging), molecular subtypes (ER/PR/HER2 status), and the presence of distant metastasis. These variables represent the strongest prognostic predictors for breast cancer. Their absence not only limits our ability to explore the heterogeneity of eGDR’s role across different risk-stratified patient groups but may also introduce residual confounding, as advanced or more aggressive tumor types may inherently correlate with poorer metabolic status. Additionally, while our multivariable models adjust for age and comorbidities, the possibility of residual confounding remains, as these variables may not have been captured with sufficient granularity. (2) The limited statistical power in our cause-specific mortality analyses is an important constraint, which is attributable to the relatively small number of death events within the survivor cohort. This insufficient event rate resulted in materially wide confidence intervals for key HRs, reducing the precision of these estimates. Consequently, these associations should be interpreted as exploratory rather than definitive evidence and validated through larger prospective cohorts with adequate mortality endpoint accrual. (3) eGDR was calculated based on a single baseline measurement, which may fail to capture the dynamic response of metabolic status during long-term follow-up and is susceptible to regression dilution bias. (4) Endpoint events were acquired from the NCHS, and delays in recording some deaths may lead to underestimated mortality risk. (5) As an observational retrospective cohort study, this research design cannot establish causation and can only report associations. Furthermore, the possibility of reverse causation remains a methodological concern. Advanced tumor progression or treatment-related comorbidities in cancer survivors may concurrently suppress eGDR and accelerate mortality, potentially confounding the observed associations. (6) Finally, while the NHANES sampling framework ensures national representativeness for the US population, the specific subsample of breast cancer survivors analyzed here (*n* = 323) is relatively small. This limited sample size, particularly in the context of diverse racial/ethnic backgrounds within the US, restricts our ability to conduct robust subgroup analyses. Consequently, caution is warranted when generalizing these findings to all US breast cancer survivors, especially across specific demographic subgroups. The applicability of our results to populations outside the US or with distinct ethnic compositions also requires further validation in future studies.

## Conclusion

Among adult women in US with a prior diagnosis of breast cancer, high eGDR is a protective factor against ACM. This association appeared to be partly explained by a potential reduction in BCSM, although this latter finding requires cautious interpretation due to its statistical imprecision and warrants validation in larger studies. Our findings suggest the profound impact of metabolic health, particularly insulin sensitivity, on the prognosis of breast cancer survivors. Future research should explore whether interventions targeting eGDR can effectively improve survival outcomes in women with breast cancer, potentially requiring personalized approaches tailored to different breast cancer stages or molecular subtypes.

## Supplementary Information

Supplementary Material 1. Description of Covariates. Figure 1 Flow chart of the Breast Cancer Survivor. Table 1 Missingness of continuous covariates.

## Data Availability

The survey data are public on www.cdc.gov/nchs/nhanes/.

## Abbreviations

ALT: Alanine Aminotransferase
AST: Aspartate Aminotransferase
BMI: Body Mass Index
BUN: Blood Urea Nitrogen
CHD: Coronary Heart Disease
CHF: Congestive Heart Failure
CI: Confidence Interval
CIF: Cumulative Incidence Functions
CVD: Cardiovascular Disease
DBP: Diastolic Blood Pressure
eGDR: Estimated Glucose Disposal Rate
HbA1c: Glycated Hemoglobin
HDL: High-Density Lipoprotein
HR: Hazard Ratio
IR: Insulin Resistance
LDL: Low-Density Lipoprotein
MI: Myocardial Infarction
NHANES: National Health and Nutrition Examination Survey
OGTT: Oral Glucose Tolerance Test
OR: Odds Ratio
RCS: Restricted Cubic Spline
SBP: Systolic Blood Pressure
Scr: Serum Creatinine
VIF: Variance inflation factor
WC: Waist Circumference
