# Supplementary material for: Association of estimated glucose disposal rate with all-cause and breast cancer-specific mortality in US breast cancer survivors: a population-based study
Source: BMC Cancer. 2025 Nov 27;26:16. doi: 10.1186/s12885-025-15340-0 (PMC12771819; doi:10.1186/s12885-025-15340-0)
Supplement: Supplementary file 1 — Supplementary Material 1. Description of Covariates. Figure 1 Flow chart of the Breast Cancer Survivor. Table 1 Missingness of continuous covariates. [file 12885_2025_15340_MOESM1_ESM.docx]

**Supplementary Material 1** Description of Covariates

All participants provided information on several demographic and lifestyle variables through survey questionnaires, including **age**, **race** (classified as Mexican American, other Hispanic, non-Hispanic White, non-Hispanic Black, or other race), **education level** (categorized as less than high school, high school or equivalent, or above high school), **PIR**（Poverty Income Ratio，calculated as: PIR =(Total Annual Gross Household Income) / (Applicable U.S. Federal Poverty Threshold)）, and **smoking status** (classified as ever smoker (defined as having smoked at least 100 cigarettes in their lifetime) versus never smoker). **Marital status** was categorized as either married or living with a partner, or living alone. Additionally, **body mass index (BMI)** was calculated using the formula: weight (kg) / height (m²) . **Vigorous work status** was defined as self-reported engagement in work involving vigorous-intensity activities that cause large increases in breathing or heart rate (e.g., carrying/lifting heavy loads, digging, or construction work) for at least 10 minutes continuously. **Moderate work activity** was similarly assessed based on self-reported engagement in moderate-intensity activities (e.g., brisk walking or carrying light loads) for ≥10 minutes continuously. Both variables were dichotomized (yes/no) and presented as frequencies and percentages (n, %) in the analysis. **Cardiovascular disease (CVD) status** was defined as a composite of self-reported physician-diagnosed conditions, including coronary heart disease (CHD), congestive heart failure (CHF), myocardial infarction (MI), stroke, or angina, as confirmed by healthcare professionals. **Depression status**, was assessed using the Patient Health Questionnaire-9 (PHQ-9), a validated tool for measuring depressive symptom severity over the preceding two weeks. Consistent with prior studies in cancer populations, a cutoff score of ≥10 was used to define clinically relevant depression. **Hypertension** was defined as meeting any of the following criteria: 1.Mean systolic blood pressure (SBP) ≥130 mmHg;2.Mean diastolic blood pressure (DBP) ≥80 mmHg;3.Prior physician-diagnosed hypertension; 4.Current use of antihypertensive medication. **Diabetes mellitus** was determined if one or more of the following were present: 1.Previous diagnosis with current use of insulin/oral hypoglycemic agents;2.Hemoglobin A1c (HbA1c) ≥6.5%;3.Fasting blood glucose (FBG) ≥126 mg/dL (7.0 mmol/L) or 2-hour glucose ≥200 mg/dL (11.1 mmol/L) during oral glucose tolerance test; 4.Random blood glucose ≥200 mg/dL (11.1 mmol/L). **Breast cancer survivors** were defined according to diagnostic criteria cited in the literature: participants reporting a prior diagnosis of breast cancer were identified as breast cancer positive, those answering “no” were breast cancer negative, while participants with many cancer diagnoses or answering “refuse” or “don't know’ to the breast cancer diagnosis question were excluded

Laboratory results were obtained from serum specimens collected during patients' visits to the mobile examination center. The serum vials were stored under appropriate frozen conditions until they were shipped to the National Center for Environmental Health for testing.


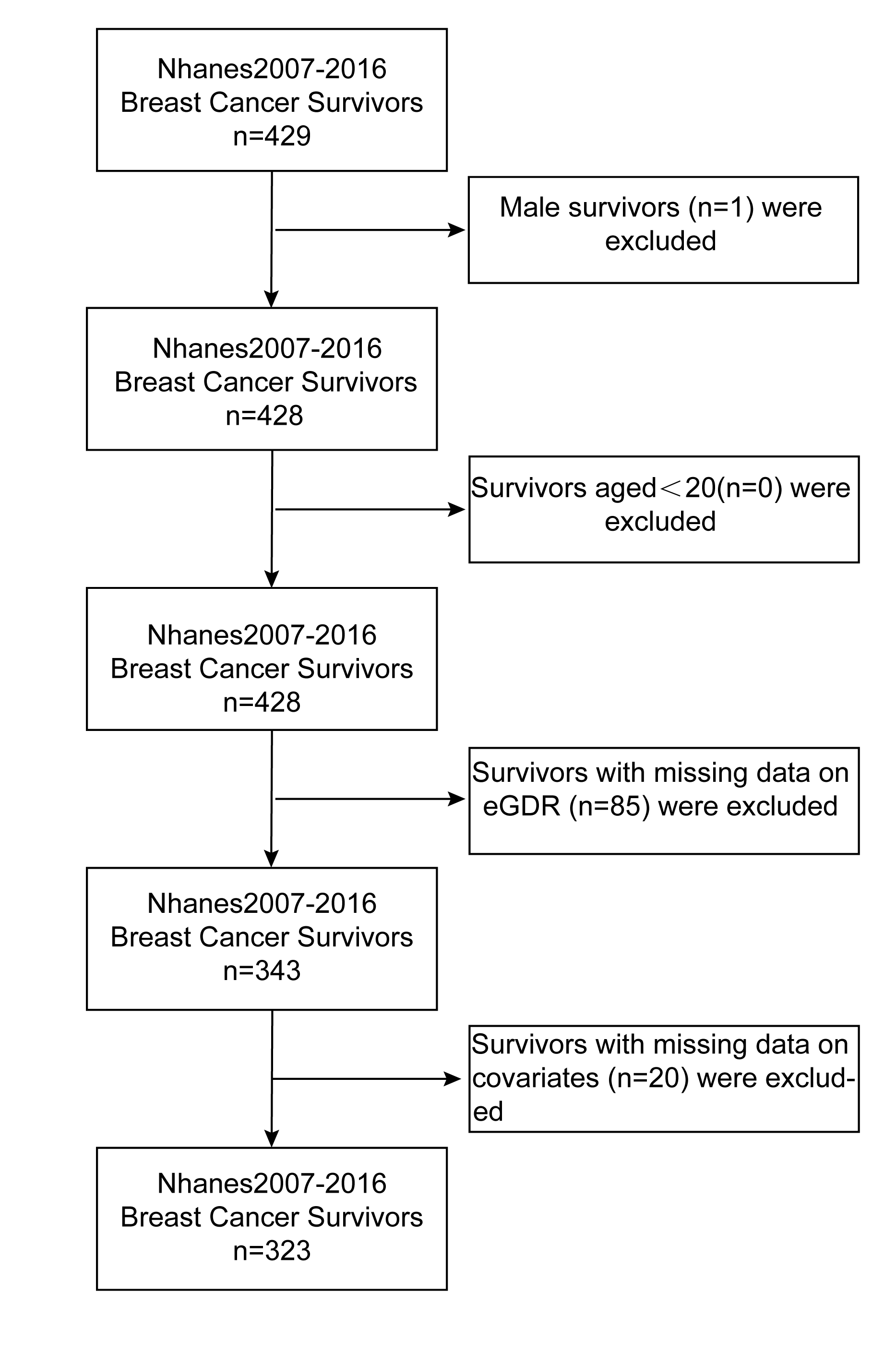


**Supplementary Figure 1** Flow chart of the Breast Cancer Survivor

**Supplementary Table 1** Missingness of continuous covariates

| Variables | Missing(n) | Total(N) | Missing rate (%) |
| --- | --- | --- | --- |
| LDL-cholesterol (mg/dL) | 6218 | 11725 | 0.530 |
| ALT,(U/L) | 197 | 11725 | 0.017 |
| AST (U/L) | 198 | 11725 | 0.017 |
| Blood urea nitrogen (mg/dL) | 189 | 11725 | 0.017 |
| Serum Creatinine (mg/dL) | 189 | 11725 | 0.017 |
| Triglycerides (mg/dL) | 200 | 11725 | 0.017 |
| BMI,(kg/m^2^) | 20 | 11725 | 0.002 |
| Family PIR | 970 | 11725 | 0.083 |
| LDL,（mg/dL） | 144 | 11725 | 0.012 |

*Excluded continuous variables: LDL-cholesterol (mg/dL) (missing rate: 53%);

*Imputed continuous variables: ALT (1.7% missing); AST (1.7% missing) ; Blood urea nitrogen (1.7% missing); Serum Creatinine(1.7% missing); Triglycerides(1.7% missing); BMI(0.2% missing); Family PIR(8.3% missing) ; LDL(1.2% missing) via the random forest algorithm

*Abbreviations: ALT: Alanine Aminotransferase; AST: Aspartate Aminotransferase; BMI: Body Mass Index; LDL: Low-Density Lipoprotein; PIR: Poverty-to-Income Ratio;
